# Evaluation of the enhanced meningitis surveillance system, Yendi municipality, northern Ghana, 2010–2015

**DOI:** 10.1186/s12879-017-2410-0

**Published:** 2017-04-24

**Authors:** Basil Benduri Kaburi, Chrysantus Kubio, Ernest Kenu, Kofi Mensah Nyarko, Jacob Yakubu Mahama, Samuel Oko Sackey, Edwin Andrew Afari

**Affiliations:** 1 Field Epidemiology and Laboratory Training Programme, Department of Epidemiology and Disease Control, School of Public Health, College of Health Sciences, University of Ghana, Legon, Accra, Ghana; 2 Ghana Health Service, West Gonja District Health Directorate, Damongo, Ghana; 3 Ghana Health Service, Northern Regional Health Directorate, Tamale, Ghana

**Keywords:** Meningitis, Epidemics, Surveillance, Evaluation, Yendi, Ghana

## Abstract

**Background:**

Meningitis is the inflammation of the meninges of the brain and or spinal cord. Global mortality rates vary from 2% to 30%. Epidemic meningitis remains a public health concern along the meningitis belt of Africa. Despite the operation of an enhanced meningitis surveillance system in Ghana, institutional mortality rates are estimated to range from 36% to 50%. In 2014, Yendi recorded 83 confirmed cases; with focal epidemics in some sub-municipals. We evaluated the system over a five-year period to find out whether it was achieving its objectives of systematic collection and analyses of data for the prevention or early detection of meningitis epidemics.

**Methods:**

We used cross-sectional design. Both qualitative and quantitative data from Yendi Municipality between January 2010 and December 2015 were collected and analyzed. The updated guidelines for evaluating surveillance systems from Centers for Disease Control and Prevention were used. Content analysis was performed on the responses of key informants. Surveillance data was analyzed using MS-Excel.

**Results:**

Fifteen healthcare workers were interviewed. For the period under evaluation, the annual incidence of meningitis ranged from 1.6/100,000 in 2012 to 62.6/100,000 in 2014. The average case fatality rate for the period was 8.3%. The system was sensitive, representative, and acceptable. The predictive value positive was 100% from 2010 to 2014 and 63.3% in 2015. Data quality was good, but timeliness of reporting was poor.

**Conclusions:**

The enhanced meningitis surveillance system in Yendi Municipality is achieving most of its objectives. However, financial constraints and poor personnel motivation pose threats to the sustainability of the system.

**Electronic supplementary material:**

The online version of this article (doi:10.1186/s12879-017-2410-0) contains supplementary material, which is available to authorized users.

## Background

Meningitis is the inflammation of the meninges of the brain. If the meninges of the spinal cord are involved, the disease state is termed Cerebrospinal Meningitis (CSM). Meningitis contributes significantly to morbidity and mortality in many regions of the world [1, 2]. There have been advancements in the diagnosis and treatment of meningitis over the years. These advances notwithstanding, mortality rates are still high.

Meningitis has both infectious and non-infectious causes. Infectious causes include: bacteria, viruses, fungi, parasites, and rickettsiae [3]. Non-infectious causes range from drugs as common as non-steroidal anti-inflammatory drugs (NSAIDs), to systemic diseases such as sarcoidosis and a host of collagen vascular disorders [3]. Neoplastic disorders, and post-vaccination complications from common vaccines such as those against rubella, rabies, and yellow fever can also cause non- infectious meningitis [3]. Viruses, non-pus producing bacteria, and some non-infectious aetiologies can cause aseptic meningitis; a unique type of meningitis with clear cerebrospinal fluid (CSF) [3].

A number of bacterial species are implicated in meningitis during the neonatal period, but about 9 in every 10 cases in the post-neonatal period are caused by *Streptococcus pneumoniae, Haemophilus influenzae,* and *Neisseria meningitides* [4]. All the three regions of northern Ghana lie completely in the meningitis belt of sub-Saharan Africa. In areas within this belt, epidemics of acute bacterial meningitis are commonly caused by different subtypes of *N. meningitides* and *S. pneumonia* [5, 6].

As at 1997, the annual non-epidemic cases of acute bacterial meningitis was estimated at 1.2 million globally with 135,000 fatalities [7]. By February 2012, WHO reported a rise in the estimated global annual fatalities to 170,000 [8]. Global mortality rates vary from 2% to 30% [9, 10]. Studies in Ghana estimate institutional  mortality rates at 36 to 50% [11]. About 10 to 20% of patients who recover from acute bacterial meningitis suffer grave sequelae such as: epilepsy, mental retardation, hearing impairment, and several related neurological deficits [4]. The estimated risk of suffering at least one complication of acute bacterial meningitis after recovery can be as bad as nearly 4 of every 10 patients; with a median risk of about 20% of such patients [4]. As would be anticipated, acute bacterial meningitis is more prevalent in developing countries and ranks as the fourth leading cause of disability in these countries [4].

Epidemic meningococcal meningitis is characterized by a sudden onset of severe headache, fever, nausea, vomiting, neck rigidity, and photophobia [12]. These primary symptoms are commonly accompanied by one or more observable neurological signs such as delirium, lethargy, coma, and convulsions [12]. These signs may not be marked in infants and a high index of suspicion would then be required for prompt diagnosis [13].

Humans are the only known natural reservoir of *N.* meningitides [14]. About one in every 10 adolescents and adults are transient carriers of many strains of *N. meningitidis*. Fortunately, most of these colonise the nasophayrnx as commensals [14]. Risk factors for contracting an infection are broadly categorized into four: environmental, demographic, socioeconomic, and host-related factors.

Environmental risk factors include hot temperatures, dusty winds, and low absolute humidity; as found in the long dry seasons of countries in the meningitis belt [14, 15]. Such conditions lead to breaks in nasopharyngeal mucosal linings and opportunistic invasion of the blood stream by *N. meningitidis* may then ensue.

Demographic factors such as mass gathering as in the cases of refugee camps, military communities, and pilgrimages promote the circulation of infectious strains among individuals. In recent history, the 1987 meningococcal outbreak at the end of a pilgrimage in Mecca illustrates the reality of this risk category [14].

Poor socioeconomic conditions result in inadequate housing and poor living standards. Overcrowding is common in these situations especially during the cold and dry harmattan season. These conditions are associated with higher incidence meningococcal disease in general [14].

Host-related factors such as compromised immunity [13] from chronic diseases such Acquired Immunodeficiency Syndrome (AIDS) and diabetes mellitus carry increased risk of contracting infection. These diseases by themselves do not pose unique threats for epidemics, except when other conducive conditions exist to initiate and facilitate spread from primary cases. Non-vaccination of susceptible populations is a serious risk factor for epidemics. A low heard immunity also poses a risk for epidemics [14]. Poorly managed respiratory tract infections can complicate into meningococcal disease epidemics [14].

The mode of spread is by droplet infection from diseased persons. Consequently, epidemics flourish in conditions where overcrowding of people is inevitable as seen in pilgrimages and refugee camps. The typical meningitis season occurs during the dry and windy harmattan season because these conditions are pertinent for the spread of the infection.

The World Health Organization (WHO) in its determination to eliminate CSM, has joined forces with other stakeholders, at the international and country level to advocate and support an enhanced surveillance system [16]. To this end, the Ghana Health Service (GHS) has in collaboration with 13 other African countries, developed a Standard Operating Procedures (SOPs) in 2004 for meningitis surveillance. An updated version was published in December 2010 by the Public Health Division of GHS to cater for lessons learnt in the 2009/2010 CSM outbreak in the country [16]. This revised SOP manual further underscores the crucial role of: the laboratory for case confirmation, readily available supplies, health personnel capacity strengthening, as well as clear operating procedures for the effective implementation of an enhanced meningitis surveillance.

From 2010 to 2013, Yendi municipality recorded a total of 19 confirmed cases of acute bacterial meningitis. Thus, an average of about five cases for each of these years. However, in 2014 alone, the municipality recorded a total of 83 confirmed cases of acute bacterial meningitis with focal epidemics in some sub-municipals. This trend of events attracts interest regarding the operation of the meningitis surveillance system in the municipality. We evaluated the system and its attributes over a six-year period to find out whether it was achieving its objectives of systematic collection and analyses of both epidemiological and laboratory data for the prevention or early detection of meningitis epidemics.

## Methods

### Study area

**Fig. 1:**
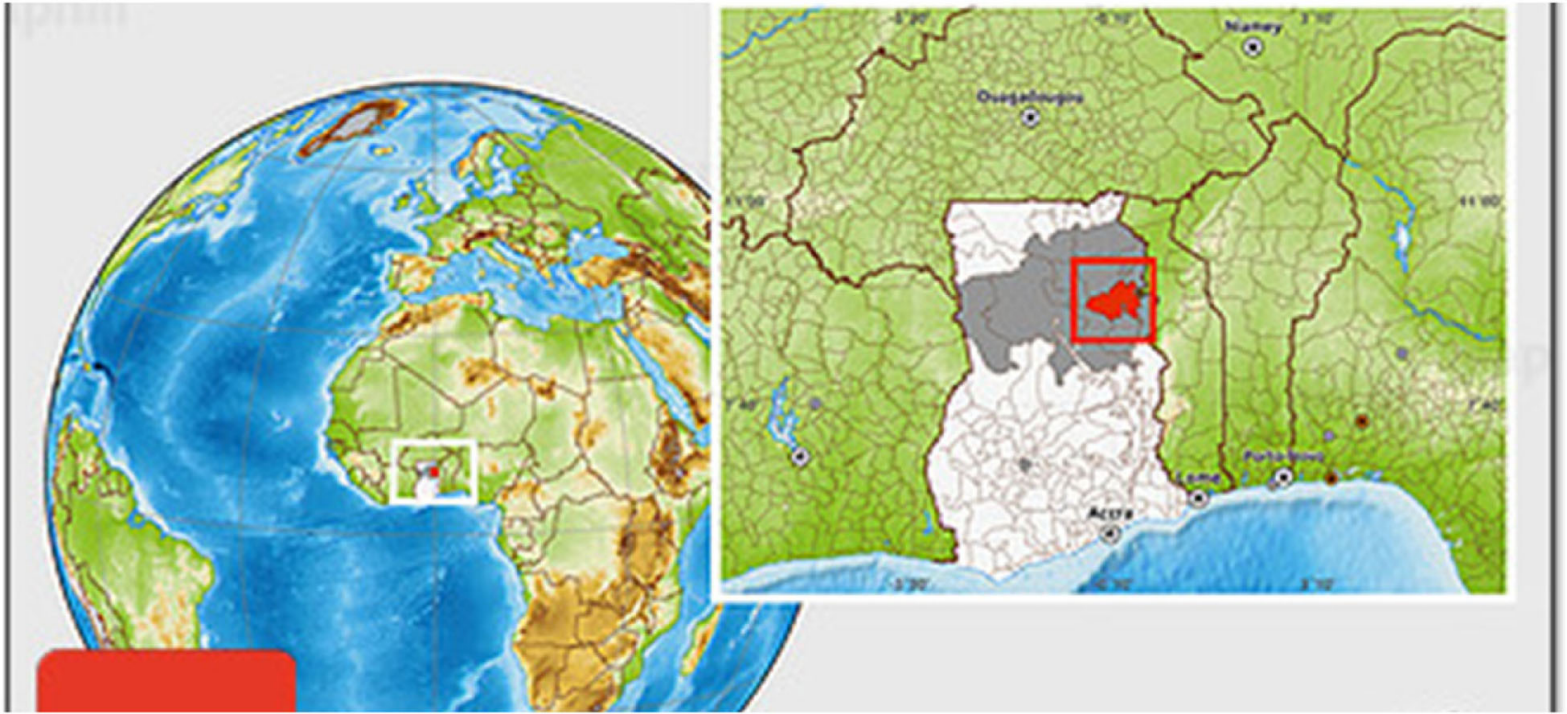
Geographical location of Yendi Municipality, Ghana. Source: google maps

The Yendi Municipality is located in the eastern corridor of the Northern Region of the Republic of Ghana between Latitude 9^o^ – 35^o^ North and Longitude 0^o^ – 30^o^ West and 0^o^ – 15^o^ East (Fig 1). The Greenwich Meridian thus passes through Yendi and other settlements in the municipality. The population of the Municipality in 2015 was about 136,434. The Dagomba ethnic group constitutes the majority. The other ethnic groups include Konkomba, Akan, Ewe, Basare, Chokosi, Hausa and Moshie.

The municipality has one hospital situated in Yendi, four (4) health centres, one (1) clinic owned by the Church of Christ, and fifteen functional Community-based Health Planning and Services (CHPS) zones. Of these CHPS zones, six (6) do not have compounds. There are five (5) main private pharmacy shops and several small chemical shops in the municipality. Drug peddling is common in the municipality.

The entire municipality lies within the African meningitis belt. Mean annual rainfall (January to December) for the Municipality is 1125 mm. Mean dry season rainfall (November to March) is 75 mm. Temperatures range between 21 °C and 36 °C and accompanied by low humidities. The vegetation is of the tree savannah type. The degraded savannah type of vegetation is found around settlements. Rampant and extensive bush burning is a significant contributor to the hot and dry climate. Such a climate is very conducive for triggering meningococcal epidemics.

### Study design

The evaluation was cross-sectional design; involving the collection of both qualitative and quantitative data. Qualitative data was collected by means of semi-structured interviewer-administered questionnaires. The following documents were used to guide the surveillance system evaluation process: the updated guidelines of the Centers for Disease Control and Prevention (CDC), for evaluating public health systems (2001), the WHO practical guidelines on the control of epidemic meningococcal disease [17], and the GHS standard operating procedures (SOPs) manual on meningitis [16]. The period under evaluation was January 2010 to December 2015.The study was conducted between December 2015 and January 2016.

### Data collection

All four Yendi sub-municipal health centers, municipal hospital, and municipal health directorate were visited. Various categories of healthcare workers were interviewed using the aforementioned questionnaires. Overall, the staff categories actively involved in the operation of the surveillance were interviewed: Technical Officers (disease control), Disease Control Officers (DCO), Physician Assistants (PA), Health Information Officers (HIO), and Biomedical Scientists (BMS). A cross section of Community-based Surveillance Volunteers (CBSVs) were also engaged in a discussion on their roles in the operation of the surveillance system. The data flow of the surveillance system was assessed and described.

Records at the four sub-Municipal health centers, Municipal Health Directorate, Regional Health Directorate, and the National Disease Surveillance Department (NDSD) of the Ghana Health Service were reviewed. These included Integrated Disease Surveillance and Response (IDSR) 1 weekly report forms, IDSR 2 monthly report forms, laboratory register of the municipal hospital, and the Zonal Public Health Reference Laboratory (ZPHRL). IDSR immediate case-based report forms, line listing forms, clinical case notes of cases captured on line listing forms, case notification forms, community based surveillance summary report forms, monitoring charts and community register for vital events were also reviewed. The reviewed records were all part of the surveillance system and spanned the period from January 2010 to December 2015.

The inspection of standard meningitis protocols supposed to be in use was done to assess their availability and ready visibility. Pieces of laboratory equipment and other relevant logistics pertinent to the meningitis surveillance system were also inspected.

### Case definitions

The following case definitions for meningitis were used: suspected, probable, and confirmed [16].

### Suspected meningitis case

*Any person with sudden onset of fever (>38.5 °C rectal or ≥ 38.0 °C axillary) and one of the following signs: neck stiffness, bulging fontanelle, convulsions, altered consciousness or other meningeal signs.*

### Probable meningitis case

*Any suspected case with macroscopic aspect of cerebrospinal fluid turbid, cloudy or purulent or with microscopic test showing Gram negative diplococci, Gram positive diplococci, Gram negative bacilli; or leukocyte count of more than 10 cells/mm* ^*3*^ *.*

### Confirmed meningitis case

*Isolation or identification of causal pathogen (Neisseria meningitides, Streptococcus pneumoniae, Haemophilus influenzae b) from the Cerebrospinal fluid of a suspected or probable case by culture, Polymerase Chain Reaction or agglutination test.*

### Data analysis

We performed both qualitative and quantitative analyses. The qualitative data gathered by means of the interviewer-administered questionnaire were analysed by directed content analysis approach [18, 19]. By this approach, the participants’ responses were systematically analysed along themes that were in keeping with the updated guidelines for evaluating public health surveillance systems as recommended by the CDC. The thematic areas included: the purpose and objectives of the system, components of the system, operational resource requirements, and the nine (9) system attributes. For each of the six years, we estimated the incidence and case fatality ratio (CFR) of acute bacterial meningitis using quantitative surveillance data retrieved from line lists.

An estimate of the amount of money spent on each suspected case of meningitis picked in the municipality was obtained by summing the personnel cost of officers involved to the costs of transport, stationary, communication, and laboratory services. The direct personnel cost was computed by summing the products of the hourly salary rates of officers involved and their respective person-hours spent on meningitis surveillance.

### Ethical considerations

Approval for this study was granted by the Ghana Field Epidemiology and Laboratory Training Programme (GFELTP); run by both the Ghana Health Service and the School of Public Health - University of Ghana. Permission was officially sought from the Regional and the Municipal Directors of Health Services to assess the surveillance system and for the use of the data. Verbal consent was obtained from interviewees without any form of coercion. Data held on computers were encrypted with a password which was made available only on a need to know basis.

## Results

### Overview

A total of 15 officers who are directly involved in the operation of the meningitis surveillance system at the various levels from the sub-municipal level to the regional level granted detailed interviews. Of these, two (2) were females. The mean age of respondents was 39.1 ± 10.7 years. For each of the four sub-municipal health facilities, there was one technical officer and one physician assistant who were in charge of the surveillance system. In all, a total of eight interviews were done at that level. At the municipal level, there were two Disease Control Officer (DCO), one Health Information Officer (HIO), and one Biomedical Scientist (BMS). Each of them was also interviewed. From the Regional level: one DCO, one BMS, and one HIO; bringing the total number of respondents to 15.

Respondents at all levels had a clear knowledge and understanding of the purpose and objectives of the system. All respondents were able to produce copies of the *standard operating procedures for surveillance and management of epidemic cerebrospinal meningitis in Ghana* as their source of written documentation of the objectives of the meningitis surveillance system. They underscored the non-negotiable role of enhanced surveillance in the early detection and confirmation of suspected cases in order that swift public health interventions would be taken to prevent outbreaks.

All respondents referred to the 1992 constitution of Ghana (Act 525 of 1996) that established the Ghana Health Service as their legal authority for data collection. They understood that the data collected would be useful if only it is analysed to produce trends. They also understood the availability of quality data as a means of providing evidence to stakeholders for purposes of obtaining support in forms such as: human resource capacity building, provision of logistics, and funds. Stakeholders as identified by respondents included: Ministry of Health, Ghana Health Service, National Health Insurance Authority (NHIA), Expanded Programme on Immunisation (EPI), GFELTP, CDC, WHO, Coalition of NGOs into Health, CBSV, Health Care Workers (HCW), traditional authorities, and opinion leaders.

The list of case definitions were available at all levels. Whereas these were boldly displayed in the form of posters at the municipal health directorate, the same was not observed at the sub-municipal health centers.

### Data communication in meningitis surveillance system

**Fig. 2:**
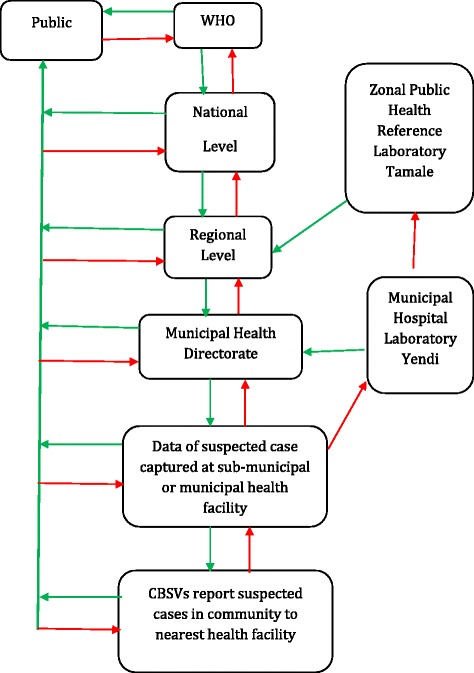
Data communication flowchart in the meningitis surveillance system

At all the levels, respondents were conversant with the flow of information for meningitis surveillance as shown in Fig. 2. However, no separate printed communication flow chart was available for meningitis. A general flow chart for all the diseases under surveillance in Ghana was available and covered meningitis as well.

The meningitis information flow started with the epidemiological reports from the community level, to laboratory reports from the municipal hospital laboratory and the Zonal Public Health Reference Laboratory (ZPHRL). Sick individuals from the community sought healthcare in a sub-municipal health facility where they could be suspected of meningitis. Other adequately informed members on health matters such as CBSVs could reported suspected cases to the facility. Sub-municipal health staff notified DCOs at the municipal level and referred suspected cases to the municipal hospital. At the municipal hospital, a Medical Officer (MO) or a nurse anaesthetist performed a lumbar puncture (LP) to sample cerebrospinal fluid (CSF). The municipal hospital laboratory, could perform the following tests: CSF glucose, cell count, Gram staining and agglutination tests. A portion of the specimen was sent to the ZPHRL for polymerase chain reaction (PCR), and culture & sensitivity tests. At the same time, the information about the case was relayed to the municipal health directorate for onward transmission to the Northern Regional Health Directorate (NRHD) and the disease surveillance department (DSD). Feedback reports from the ZPHRL were sent in the reverse direction through NRHD, YMHD, sub-municipal health facilities and finally back to the community. The NRHD fed the national DSD with information, which in turn fed WHO. During outbreaks or suspected outbreaks, the public and key stakeholders received updates of information at every stage of the investigation.

### Components of the meningitis surveillance system

The entire population of the municipality; males and females of all ages, was under surveillance. DCOs and HIOs were responsible for collecting primary data on suspected cases. They collected and reported these data on daily, weekly, and monthly bases. For any given suspected case, the sub-municipal DCO completed a case notification form to notify municipal officers. Staff of sub-municipal health centers did not have the capacity to perform lumbar punctures (LPs). Suspected cases were therefore referred to the municipal hospital at Yendi where LPs were performed to sample CSF. Each specimen was accompanied by a completed meningitis case investigation form. Line listing forms were available for capturing multiple cases. Additionally, contact tracing and follow-up forms were available for tracing patient contacts in the communities.

A functional data validation team was in place at the municipal health directorate. This ensured that quality data is generated. Data quality validation was done by cross examining all hard copies of reporting tools for completeness and consistency before entering them onto the District Health Information Management System-2 (DHIMS-2) database. Upon entry of data onto the DHIMS-2, care was taken to ensure completeness and consistency of information before logging out. At the regional level, data was entered into Epi-info7 and check codes put in place to ensure proper validation. Physical checks were done and phone calls made to reporting districts whose data had inconsistencies for reconciliation. The northern regional weekly epidemiological bulletins also served to assist in data validation.

Hard copies of surveillance data were kept in files. Soft copies were saved in office computers and the DHIMS-2 under passwords. Additionally, data was stored on external hard drives. The DCOs and HIOs analysed the data using MS-Excel, Epi-info, and SPSS. Graphs, tables, and spot maps were generated.

### Surveillance system resource requirements

The meningitis surveillance system has no separate budgetary allocation for its operation. It is part of the integrated disease surveillance system. It therefore shared resources allocated to all surveillance activities.

At the municipal level, six persons were directly involved in the meningitis surveillance system. For the year 2015, 30 suspected cases of meningitis were reported. The total personnel cost was estimated at GHC 137.48 per case. Table 1 shows a summary of estimations of the cost of handling each case.

**Table 1:** Personnel Cost Estimation for Meningitis Surveillance, Yendi Municipal Health Directorate, 2015

Designation of officer	Hourly salary rate (GH¢)	Person-hours per case	Cost of service per officer (GH¢)
MDHS	36.25	0.75	27.19
MO	22.50	0.50	11.25
BMS	11.25	1.00	11.25
DDCO	10.62	2.50	26.55
STO	8.75	4.00	35.00
TO	6.56	4.00	26.24

**Table 2:** Incidence and Case Fatality Rate of meningitis, Yendi Municipality, 2010–2015

Year	Mid-Year Population	No. of Cases	No. of Deaths	Incidence/100,000	Case Fatality Rate (%)
2010	118,260	6	2	5.1	33.3
2011	121,690	8	1	6.6	12.5
2012	125,220	2	0	1.6	0.0
2013	128,850	3	1	2.3	33.3
2014	132,589	83	5	62.6	6.0
2015	136,434	19	1	13.9	5.3

Estimated costs of laboratory services (CSF glucose, cell count, Gram staining, and pastorex) at the municipal hospital was GH¢ 26.00 per case. Estimates of other routine costs were: communication - GH¢ 7.0, stationary - GH¢ 2.5, and the transportation of samples to the ZPHRL- GH¢ 23.00. These non-personnel costs summed up to GH¢ 58.50 per case. A grand total of GH¢ 195.98 (USD 52.96) was spent on each suspected case that underwent investigation.

Other resources required for meningitis surveillance activities included costs of training, logistics, and allowances of health personnel working on this system. Costs of Medical supplies, routine travel, information management, and case management were however excluded from the above estimates.

### Usefulness of the meningitis surveillance system

The operation of the meningitis surveillance system in Yendi municipality was found to be very useful. From epidemiological and laboroatory data generated over the period, the overall case fatality rate was 8.3%. Table 2 depicts the annual CFR for the period evaluated. In 2014, some sub-municipal areas exceeded their epidemic thresholds even though the municipal as a whole was at the alert stage. Focal epidemics were therefore picked up by the system. As a result, reactive vaccinations within those affected areas and their neighbouring communities were carried out.

The meningitis surveillance data are also used to help prepare annual meningitis epidemic preparedness plans. These data were also useful in making realistic estimations of resource requirements for prevention and containment of any epidemic. Stakeholders also depended on these data to make decisions on the nature and quantum of support that should be given for the operation of the system.

### System attributes

#### Simplicity

The system was rated as simple because of the clarity of its case definitions and the ease with which cases could be captured using easily detectable symptoms and observable signs. It took less than 30 min to collect data required to fill the new and separate meningitis case investigation form. The demographic and exposure information were easy to understand and record. Though there were multiple levels of reporting, all the 15 respondents demonstrated a good understanding of the scope of their respective roles in the system. Sequential relay of information from the sub-municipal through the YMHD and NRHD to the national level was effective.

#### Flexibility

The system was found to be flexible because it was well integrated with other surveillance systems. Additionally, modifications in its operations did not cause any setbacks. For example, the meningitis case investigation form was introduced in 2012 to replace the case based surveillance reporting form which catered for 5 other diseases under surveillance namely: cholera, diarrhoea with blood/shigella, measles, viral haemorrhagic fever, and yellow fever.

#### Stability

The systems was seen to be fairly stable partly because it is fully integrated with the other diseases under surveillance. The energy crisis that hit the country for the past 3 years posed challenges to the information management systems and communication due to rampant and unscheduled power outages. This notwithstanding, surveillance activities went on satisfactorily because on some occasions, power backups such as generators were used to mitigate the crisis. Other routine challenges with logistics, transport, and communication were also apparent during the evaluation.

#### Representativeness

The surveillance system was representative in person and place over the time period evaluated. The surveillance system collected data all year round with intensification of active case searches during the dry season and epidemics. It was noted that for the period under evaluation, 89.4% of the cases were recorded in the first quarter of the year; and none in the last quarter. The latest recorded case was in week-36 of 2015.

Over the six-year period all persons were under surveillance. Cases were reported from: both sexes, all ages, ethnicities, religions, occupations, and socioeconomic backgrounds. Cumulatively, 61.4% of cases were females.

In the four (4) years that preceded the epidemic, a total of 19 cases were recorded. These cases originated from all sub-municipals, they included males and females, and their ages ranged from one (1) year to 45 years. This representative distribution of cases was still true when individual years within the pre-epidemic period were considered. For example in 2011, when eight (8) cases were recorded, these were distributed across seven different sub-municipalities, with five females, and ages ranging from one (1) to 45. Similarly, with only three recorded cases in 2013, three sub-municipalities were represented, two of the three cases were females, and their ages were 2, 8, and 30 years.

#### Acceptability

The meningitis surveillance system was very acceptable among all stakeholders. All 17 reporting units of the Yendi municipality reported to the municipal health directorate. All 26 districts of the northern region also submit reports to the regional health directorate as evidenced in the weekly epidemiological bulletins. Community members, CBSVs, health care professional of all categories, and patients all cooperated in the reporting and investigation of suspected cases. At the municipal level, LP rates have been consistently100% over the 6 year period under evaluation. The participation of CBSVs was however passive. They attributed this development to poor motivation.

#### Predictive value positive (PVP)

At the level of case detection, the predictive value positive was100% for each of the five (5) years from 2010 to 2014. Thus, all the suspected cases turned out to be confirmed as positive cases. However, in 2015, 19 of the 30 reported suspected cases turned out to be positive. This gave PVP of (19/30)*100% = 63.3%.

At the level of epidemic detection, the PVP was also 100%; as the only epidemic identified by the surveillance system within the study period (in 2014) turned out to be a true epidemic.

#### Sensitivity

The system was assessed qualitatively and found not to be very sensitive at the level of case detection because it was not able to detect many cases from 2010 to 2014. However, at the level of outbreak detection, it was sensitive enough to detect focal outbreaks in some sub-municipal areas in 2014.

#### Timeliness

Timeliness was assessed to be poor. It was assessed at three stages: timeliness of patient reporting to health facilities, timeliness of delivery of CSF to ZPHRL, and timeliness of the feedback from ZPHRL to municipal hospital and other stakeholders.

It took an average of three days (3 ± 0.8 days) for symptomatic patients to report to the nearest health facility within the municipality. Delivery of CSF samples from YMHD to the ZPHRL took three days on the average (3.0 ± 1.6 days). Sometimes samples could delay up to seven days. It took an average of five days (4.7 ± 2 days) to receive feedback from the ZPHRL.

#### Data quality

The quality of data generated in the municipality did not have major issues. On cross examination, information on hard copies of line listing forms and case investigation forms from the reporting units were mostly consistent with that found at the sub-municipal records. A few data reconciliations had to be done on the geographical origin of some suspected cases.

Case investigation forms were largely complete. However, in about 24% (32/132) of cases, the immunisation histories could not be obtained. The LP rate was 100% and all such cases had records of laboratory investigations performed on CSF samples. The municipal data as captured in their records and DHIMS-2 were also largely consistent with those available at the NRHD. A feedback of five additional confirmed cases were yet to reach the YMHD. The NRHD was also yet to update the number of suspected cases for 2015, from 23 to 30 as at the time we reviewed their records.

## Discussion

The enhanced meningitis surveillance system, as run in the Yendi Municipality is largely effective despite human resource and financial constraints. It is achieving the key surveillance objectives of early detection and prevention of epidemics. Even in the case of the focal epidemics within the municipality in 2014, the response was early enough to avert a major epidemic. The response took the forms of sensitization and health education of the general public, and a reactive vaccination exercise. The public was educated on how to recognise signs and symptoms of meningitis, the facilities to report to, and how to avoid the spread of the disease. This was done by the epidemic response teams of the municipal and region using the mass media, community durbars, schools, and places of worship. Confirmatory PCR results from the ZPHRL indicated the causative organism was Nm W. Residents of the municipality were encouraged to patronise the reactive vaccination exercise against Nm W. This exercise started within a week of the outbreak detection. These interventions helped to contain the spread of the outbreak.

The system was found to be simple, flexible, and highly acceptable to all stakeholders. It was fairly stable despite the energy and economic crisis the country had experienced over the period under review. The multiple storage forms of data that were in place provided backups should one storage form get lost or corrupted. The attainment of the remaining system attributes is largely contingent on performance of these preceding four surveillance system attributes. This assertion is very much in keeping with findings of similar evaluation studies of dengue fever in Taiwan [20] and of meningitis in the Talensi district in the Upper East Region of Ghana [21].

The high level of acceptability had a positive influence on data quality. Any evidence of effective operation of the system will be demonstrated by the reliability of the data generated. This in turn is not possible if the system is not very representative in time, person, and place. Cases of meningitis were reported from all sub-municipals and these covered both males and females of all age groups. Of the 132 reported suspected cases for the period under review, majority (61.4%) were females. This finding is also consistent with the general literature that espouses a higher prevalence among females. Females are thought to be at a higher risk of secondary attack because they mostly take care of family members who become primary cases. This was observed in the evaluation of the meningitis surveillance systems of Burkina Faso and Mali [22]. Two in every three cases were aged 29 years and younger. This gives further credence to the ascription of the most at-risk age group to those between 2 to 29 years [22]. All persons from neighbouring communities outside the municipality, and temporary residents who are in transit for socioeconomic reasons were also considered as part of the target population. These were captured as imported cases. They accounted for nearly a quarter (23.1%) of the 121 confirmed cases within the period under evaluation.

The PVP of 100% could be attributed to a strict adherence to the standard case definition for a suspected case. Thus, meeting the criteria for a suspected case invariably led to the confirmation of acute bacterial meningitis. However, the system could have been missing cases as a result of this strict adherence to the case definition. The PVP reduced to 63.3% in 2015 because a broader case definition was adopted. This broader case definition differed only in one respect from the standard one: it did not require all suspected cases to present with fever or honestly disclose a history of fever. The reason was that, it is common practice in Ghana for patients to resort to NSAIDs for self-medication as a first option when they develop a fever. They would also try treating themselves for malaria because it is still the commonest cause of fever among Ghanaian patients. As a result, they may subsequently present to the health facility without fever. Some may also fail to disclose a history of fever for which they self-medicated for fear of being reprimanded by clinicians. The recognition of these common patient behaviours informed the reduced importance placed on fever in suspecting a meningitis case in 2015. Furthermore, a higher index of suspicion within the population and among clinicians, informed by the focal epidemics that hit parts of the municipality in 2014, also contributed to increased reporting of suspected cases and hence the reduced PVP. The relatively high prevalence of endemic meningitis in this municipal probably contributed to these findings which stand in contrast to those of the Talensi district as reported by Apanga and Awoonor-Williams [21].

Even though the YMHD promptly alerted the NRHD of suspected meningitis cases, this did not translate to prompt laboratory investigation of CSF specimen for a number of reasons. Firstly, the YMHD had some difficulties delivering specimen of CSF to the ZPHRL due to transportation challenges. Secondly, investigation and feedback from the ZPHRL could delay up to a week on some occasions. Unlike the challenge of distance cited by Apanga and Awoonor-Williams [21] as mainly responsible for delays in feedback, Yendi Municipality is about one hour drive from the ZPHRL in Tamale. Thus, apart from distance, other challenges to do with personnel and logistics are at play as indicated by some of the managers interviewed. Delays in feedbacks tend to undermine the whole essence of the surveillance system. The reason being that, prompt public health actions that are key to averting epidemics may be delayed as a result.

The Nm W was the causative organism identified by the ZPHRL at Tamale. In 2012, the same organism was responsible for causing outbreaks first in the Upper West region [23] and then in the Upper East region [24]. Given that this same organism had caused two successive outbreaks in two of the neighbouring regions, the public health community in Ghana was not surprised because routine laboratory surveillance kept recording increasing numbers of this organism across the three northern regions of the country. However, delays in laboratory confirmation of cases during non-epidemic periods contribute to delays in outbreak detection. This situation could be improved by tackling two main problems: ensuring readily available means of transport to deliver specimen speedily to the ZPHRL, and boosting the logistic capacity of this laboratory to perform investigations speedily. Equipment breakdown is one recurring reason for laboratory delays and so the culture of plan preventive maintenance should be enforced particularly at the ZPHRL.

The importance of the laboratory in the operation of the enhanced meningitis surveillance has been demonstrated severally [25]. Analyses of laboratory data before 2010 indicated that *N. meningitides* strain A was mostly responsible for large meningococcal outbreaks in Ghana. Similar findings were made in countries along the African meningitis belt [16]. This necessitated a policy change by which Men A vaccines were substituted for previous ones in mass vaccinations. Consequently, the prevalence of this strain has reduced. No Meningitis A epidemics have occurred in Ghana since the mass vaccination was carried out 2012.

Unlike funded programmes such as Tuberculosis, HIV/AIDS, Malaria, and Maternal health, meningitis surveillance has no dedicated budgetary allocation to ensure unfettered execution of its activities. Its reliance on the common budget allocated for the integrated surveillance activities poses difficulties of motivating non-salaried players in the chain of reporting such as the CBSVs. Their place at the very beginning of the reporting pathway is crucial to early detection of suspected cases for subsequent investigations at our health facilities. Our interaction with a cross-section of 17 CBSVs of Gnani sub- municipality revealed that they no longer feel a part of the system as they did about eight years before. Hitherto, they had opportunities of attending regular training workshops and earning some allowances. They also indicated that, they and their families no longer enjoy express services at health facilities. In the absence of all these incentives, a good number of them have abandoned their duties, and the remaining ones do not do any more than passive reporting. Passive reporting on the part of these CBSV contributed to the poor timeliness of case reporting from the community to health facilities. This is a worrying development because, the foundation of the surveillance hinges on these CBSVs.

## Study limitations

Some limitations of the study include the following; Clinical case notes for periods when there were no outbreaks, were sampled and reviewed. As such, it is possible some cases were missed due to the sampling process.

Also the review required the interviewees recalling events over the past five years, there is therefore likely to be a recall bias, however, this was minimized since frontline workers were interviewed.

## Conclusions

The meningitis surveillance system operated by the Yendi municipality is achieving most of its objectives even in the face of systemic challenges that border largely on inadequate finances. The system was found to be simple, flexible, and acceptable. It was fairly sensitive, representative, and stable. The data quality was good and the PVP was high. However, timeliness of case reporting from communities to health facilities and specimen collection at municipal hospital were poor. There was low morale among CBSVs resulting in passive case reporting.

The WHO, coalition of NGOs into health, and other donor partners should consider increasing the funding support for the integrated surveillance activities in order to consolidate the gains made this far. The low morale of our indispensable CBSVs could be improved if managements of the health centers and municipal hospital could renew their commitment to engage them regularly, and also assist them access health care services speedily for themselves and immediate family members. GHS should also consider training physician assistants on how to perform lumbar puncture in order to improve timeliness of CSF collection.

## Additional file

Additional file 1: Meningitis Surveillance Data Yendi 2010-2015. (XLSX 36 kb)

## Abbreviations

AIDS: Acquired Immunodeficiency Syndrome
BMS: Biomedical Scientist
CBSVs: Community Based Surveillance Volunteers
CDC: Centers for Disease Control and Prevention
CHPS: Community-based Health Planning and Services
CSF: Cerebrospinal Fluid
CSM: Cerebrospinal Meningitis
DCO: Disease Control Officers
DHIMS: District Health Information Management Systems
EPI: Expanded Programme on Immunization
GFELTP: Ghana Field Epidemiology and Laboratory Training Programme
HCW: Health Care Workers
HIO: Health Information Officers
IDSR: Integrated Disease Surveillance and Response
LP: Lumbar Puncture
MO: Medical Officer
NDSD: National Disease Surveillance Department
NRHD: Northern Regional Health Directorate
NSAIDs: Non-Steroidal Anti-inflammatory Drugs
PA: Physician Assistant
SOPs: Standard Operating Procedures
WHO: World Health Organization
YMHD: Yendi Municipal Health Directorate
ZPHRL: Zonal Public Health Reference Laboratory.
